# ACP-BC: A Model for Accurate Identification of Anticancer Peptides Based on Fusion Features of Bidirectional Long Short-Term Memory and Chemically Derived Information

**DOI:** 10.3390/ijms242015447

**Published:** 2023-10-22

**Authors:** Mingwei Sun, Haoyuan Hu, Wei Pang, You Zhou

**Affiliations:** 1 Key Laboratory of Symbol Computation and Knowledge Engineering of Ministry of Education, College of Computer Science and Technology, Jilin University, Changchun 130012, China; sunmw19@mails.jlu.edu.cn (M.S.); huhy22@mails.jlu.edu.cn (H.H.); 2 School of Mathematical and Computer Sciences, Heriot-Watt University, Edinburgh EH14 4AS, UK; w.pang@hw.ac.uk; 3 College of Software, Jilin University, Changchun 130012, China

**Keywords:** anticancer peptides, bidirectional long short-term memory, chemical information

## Abstract

Anticancer peptides (ACPs) have been proven to possess potent anticancer activities. Although computational methods have emerged for rapid ACPs identification, their accuracy still needs improvement. In this study, we propose a model called ACP-BC, a three-channel end-to-end model that utilizes various combinations of data augmentation techniques. In the first channel, features are extracted from the raw sequence using a bidirectional long short-term memory network. In the second channel, the entire sequence is converted into a chemical molecular formula, which is further simplified using Simplified Molecular Input Line Entry System notation to obtain deep abstract features through a bidirectional encoder representation transformer (BERT). In the third channel, we manually selected four effective features according to dipeptide composition, binary profile feature, k-mer sparse matrix, and pseudo amino acid composition. Notably, the application of chemical BERT in predicting ACPs is novel and successfully integrated into our model. To validate the performance of our model, we selected two benchmark datasets, ACPs740 and ACPs240. ACP-BC achieved prediction accuracy with 87% and 90% on these two datasets, respectively, representing improvements of 1.3% and 7% compared to existing state-of-the-art methods on these datasets. Therefore, systematic comparative experiments have shown that the ACP-BC can effectively identify anticancer peptides.

## 1. Introduction

Cancer is a severe disease causing a considerable number of deaths globally [1]. It is characterized by uncontrolled and aberrant cell growth, rapid proliferation, or invasion into the human body, constituting formidable illnesses [2]. According to diagnostic and reporting data from international cancer research institutions [3], there have been over 19.3 million new cases of cancer worldwide, resulting in approximately 10 million deaths by the year 2020. The global cancer burden is expected to be 28.4 million cases in 2040. Conventional cancer treatment methods include radiation therapy, chemotherapy, surgery, as well as targeted drugs and immunotherapy [4]. However, commonly employed techniques such as radiation therapy and chemotherapy have detrimental effects on healthy cells, exhibiting noticeable side effects, low success rates, and carrying the risk of relapse. Additionally, these methods are financially burdensome [5]. Although targeted drug therapies do not harm normal cells, they may still induce certain side effects such as skin inflammation, fatigue, nausea, and vomiting [6]. Furthermore, traditional drug treatments often lead to the development of drug resistance in cancer cells [7]. Therefore, there is an urgent need to develop novel anticancer drugs that can effectively inhibit the rapid proliferation of cancer cells.

The emergence of anticancer peptides has opened up new avenues for cancer treatments. Anticancer peptides are naturally occurring small-molecule peptides composed of 5–40 amino acids, known for their high biocompatibility and low toxicity [8]. The identification and characterization of these peptides in tumor biology experiments are time-consuming, labor-intensive, costly, and challenging. Therefore, there is an urgent need to seek rapid and accurate methods for identifying anticancer peptides. Anticancer peptides have demonstrated promising therapeutic effects in cancer treatments and have been recognized as potential anticancer agents [9,10,11]. Currently, a growing number of anticancer peptides have been identified and validated from protein sequences through clinical experiments. Peelle et al. [12] showcased the effectiveness of intracellular protein scaffold-mediated random peptide libraries using mammalian cell phenotypic screening methods. Norman et al. [13], on the other hand, employed genetic approaches to select and inhibit bio-pathway peptides. However, the use of these identification methods is time-consuming, labor-intensive, costly, and challenging. Hence, there is an urgent need to explore rapid and accurate methods for identifying anticancer peptides [14].

Many computational techniques have been widely applied in the field of bioinformatics to solve various problems [14]. In the recognition of anticancer peptides (ACPs), machine learning has demonstrated absolute advantages and prospects [15,16,17,18,19]. Over the past few years, a series of traditional machine learning methods have been proposed for ACPs identification. These traditional methods require manual design of features to classify protein sequences. As a result, various methods for extracting effective features have emerged, among which the support vector machine (SVM) model is the most commonly used method. Tyagi et al. [9] first proposed the use of machine learning models for ACPs identification. They developed the AntiCP model, which selected amino acid composition (AAC) [20], split AAC (using N-terminal and C-terminal residues), dipeptide composition (DPC) [21,22], and binary profiles feature (BPF) [22] as features of peptide sequences. These features were used as inputs to an SVM classifier to distinguish ACPs from non-ACPs sequences. Hajisharifi et al. [23] proposed two SVM-based methods for ACPs identification. The first method employed pseudo-amino acid composition (PAAC) [24,25,26,27,28] to extract combination features of six physicochemical properties of amino acids. The second method extracted features from peptide sequences using the core local alignment technique and utilized SVM for binary classification. Vijayakumar et al. [29] developed the ACPP model, which selected amino acid distribution measurement-based features and centroid composition information as features, combined with an SVM model for ACPs identification. Chen et al. [30] developed the iACP model, which utilized g-gap dipeptide composition (g-gap DPC) for feature extraction of peptide sequences and employed radial basis function (RBF) kernel supported SVM for classification. The Random Forest (RF) [31] model is also a commonly used method for identifying ACPs. Manavalan et al. [2] developed the MLACP model, which selected AAC, DPC, ATC, and physicochemical properties of residues for feature extraction, and utilized SVM and RF classifiers for ACPs recognition. Akbar et al. [32] proposed the iACP-GAEnsc model, which selected g-gap DPC, reduced amino acid alphabet composition (RAAAC), and PAAC based on amino acid hydrophobicity for feature extraction, and applied a combination of SVM, RF, probability neural network (PNN), generalized regression neural network (GRNN), and k-nearest neighbors (KNN) classification models for ACPs identification. Wei et al. [33] proposed a PEPred-Suite model based on RF, which further improves the feature representation of ACPs to predict anticancer peptides. Boopathi et al. [34] proposed an mACPpred model, which uses seven specific types of encoding features, including AAC, DPC, composition-transition-distribution (CTD), quasi-sequence-order (QSO), amino acid index (AAIF), binary profile (NC5), and conjoint triad (CTF) to represent a peptide sequence and cooperate with an SVM model to predict ACPs. Li et al. [35] selected AAC, PAAC, and grouped amino acid composition (GAAC) features to construct a low dimensional feature model to identify anticancer peptides. Xu et al. [36] proposed a sequence-based hybrid model that transformed polypeptides into feature vectors using g-gap DPC and employed SVM and RF as classifiers. Schaduangrat et al. [37] introduced the ACPred model, which selected AAC, DPC, PAAC, amphiphilic pseudo amino acid composition (Am-PAAC), and physicochemical properties as features of peptide sequences, and used SVM and RF for ACPs identification. Meanwhile, Wei et al. [38] developed a sequence-based anticancer predictor called ACPred-FL, which employed a two-step feature selection technique and selected peptide length, BPF, overlap property feature (OPF), twenty-one-bit feature (TOBF), CTD, AAC, g-gap DPC, and adaptive skip dipeptide composition (AKDC) as seven representation methods of features.

However, with the rapid development of the big data era in recent years, there has been an explosive increase in biological big data, making traditional machine learning algorithms inadequate for handling complex and diverse data. Deep learning methods, known for their ability to efficiently process unstructured data, have been widely applied in the field of bioinformatics. An increasing number of deep neural network models have been employed for ACPs recognition [19,39,40]. Wu et al. [41] developed PTPD, which utilized word2vec to represent k-mer sparse matrixers [42] and employed convolutional neural networks (CNN) for ACPs recognition. Yi et al. [43] proposed ACP-DL, which selected BPF, a reduced amino acid alphabet, and the k-mer sparse matrix as features, and applied long short-term memory (LSTM) models for ACPs prediction. Cao et al. [44] presented the DLFF-ACP model, using AAC, DPC, k-spaced amino acid group pairs (CKSAAGP), and Geary as features, and integrating deep learning and multi-view feature fusion for ACPs identification. Ahmed et al. [40] recently developed APC-MHCNN, a computational model for predicting anticancer peptides that utilizes a multi-headed deep CNN. In their study, they selected sequence, physicochemical, and evolutionary features as inputs to the model. By employing a deep learning approach, the ACP-MHCNN demonstrated promising performance in peptide prediction. Similarly, Sun et al. [45] introduced ACPNet, a novel framework for identifying anticancer peptides. ACPNet incorporates peptide sequence information, physicochemical properties, and self-encoding features into its architecture. The model employs fully connected networks and recurrent neural networks to achieve accurate ACPs classification. Wang et al. [46] proposed CL-ACP, which introduces the anticancer peptides secondary structures as additional features and uses a combined network and attention mechanism to predict anticancer peptides. Chen et al. [47] proposed ACP-DA, which integrates BPF and k-mer sparse matrix features to represent peptide sequences and uses data augmentation to improve the predictive performance of anticancer peptides. Rao et al. [48] proposed the ACP-GCN model, which leverages one-hot encoding and graph convolutional networks (GCN) to predict anticancer peptides. By utilizing the unique characteristics of peptide sequences and considering their structural relationships through GCN, the ACP-GCN model achieves high accuracy in ACPs identification. Zhu et al. [49] developed the ACP-check model, which uses LSTM networks to extract time-dependent information from peptide sequences for anticancer peptides to be identified effectively. You et al. [50] fused the sparse matrix features of BPF and the k-mer sparse matrix to construct a new bidirectional short-term memory network, which achieves the prediction of anticancer peptides through two sets of dense network layers. The aforementioned studies demonstrate significant advancements in the field of computational peptide-based cancer research. The development of these computational models provides valuable tools for predicting and identifying potential anticancer peptides, thereby facilitating the discovery of novel therapeutic agents for combating cancer.

Although the above studies have made some progress, there is still room for improvement. For instance, the above methods only consider the information derived from the amino acid primary sequence and do not take into account the spatial structural information of amino acids. In this study, we propose a novel deep learning model for ACPs prediction called ACP-BC, which is an end-to-end model that combines sequence and chemical information to predict whether a protein sequence is an ACP. The features extracted by ACP-BC are divided into three channels. The first channel extracts features through a three-layer bidirectional long short-term memory (Bi-LSTM) [51,52]. The original sequence is first mapped to a 256-dimensional vector through an embedding layer fused within the model, and then input into the Bi-LSTM for feature extraction. The second channel utilizes information from the chemical bidirectional encoder representation transformer (BERT) [53,54]. We convert the entire sequence into the form of a chemical molecular formula and then use the Simplified Molecular Input Line Entry System (SMILES) [55,56,57] to further simplify it. This SMILES-encoded sequence is input into a pre-trained BERT model for fine-tuning, resulting in abstract features at a deeper level. The third channel consists of manually crafted features known to be effective, including BPF, DPC, PAAC, and k-mer sparse matrix features. These four types of features are fused together to collectively extract features at different levels of an amino acid sequence.

**Figure 1:**
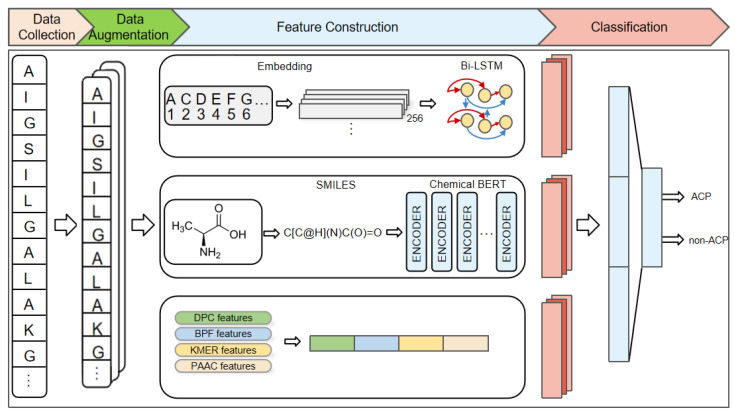
An overview of the proposed ACP-BC model. Input the original peptide sequence, perform data augmentation, followed by feature extraction from three channels: Bi-LSTM, pre-trained BERT, and handcrafted features. The features from these channels are concatenated and used for classification via a fully connected layer. The trained model is then evaluated.

Our proposed method can be divided into three steps, as shown in Figure 1. Firstly, data collection is conducted by inputting the given peptide sequences and expanding the data using two combination methods. Then, feature construction is carried out, and peptide sequences are processed by the previously mentioned Bi-LSTM, pre-trained BERT, and feature engineering methods, respectively, to extract features from the three channels. Finally, feature classification connects the features of these three channels and uses a fully connected layer to classify peptide sequences, train the model, and evaluate the trained model. The experimental results indicate that our designed model can better extract deep features, utilizing better representation of peptide sequences and a reasonable model structure. ACP-BC can achieve high accuracy and can be more effectively applied to ACPs prediction.

## 2. Results

### 2.1. Analysis of Amino Acid Composition

**Figure 2:**
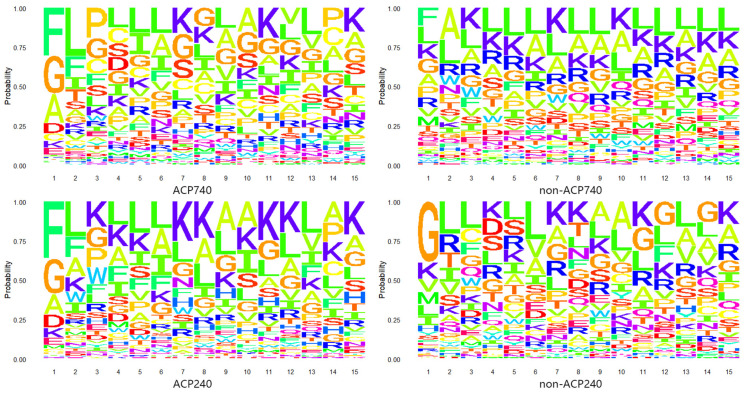
Anticancer peptides probability logo graph. In the ACP740, non-ACP740, ACP240, and non-ACP240 datasets, the positional preference of amino acid residues in anticancer peptides and non-anticancer peptides.

Anticancer peptides (ACPs) are small peptides typically composed of 5–40 amino acids [8]. To investigate the positional preference of amino acid residues in ACPs and non-ACPs, we extracted the first 15 N-terminal residues from two benchmark datasets, ACP740 and ACP240 [43], and created probability logo plots [58], as shown in Figure 2. In the plots, larger letters indicate that the amino acid is more frequently present at that position. Preliminary observation shows that the letter F represents that Phenylalanine exists more frequently at the N-terminal of ACPs, while the letters G, A, and D represent glycine, alanine, and Aspartic acid, respectively, which also occupy the majority of the N-terminal, but their physical and chemical properties are very different. At the second position, the letter L stands for Leucine and the letter F is the most common. They have similar properties. At other positions, the letter K represents lysine and the letter L is frequently present, but also appears in non-ACPs. Although there are differences between ACPs and non-ACPs, there is also great variability among ACPs. Therefore, distinguishing ACPs from non-ACPs through the positional preference of amino acid residues remains a challenging issue.

### 2.2. Parameters of ACP-BC

In the experiment, numerous hyperparameters require manual configuration. We conducted experiments to determine the optimal selection of several key hyperparameters, which have a significant impact on the results. These hyperparameters include the data augmentation factor R (1.0, 2.0), parameter C (128, 256, and 512) for the number of neurons in the LSTM hidden layer, and parameter D (128, 256, and 512) for the number of neurons in the embedded layer. R represents the multiplier for data augmentation, where R = 1.0 means that the training dataset is augmented once and added to our training set. In terms of quantity, the entire training set is expanded to twice the original size. The number of neurons D in the embedding layer is also a parameter that significantly influences the results, as it represents the length of the vector encoding for each amino acid residue in the original peptide chain. To explore the best effect of key hyperparameter combinations, the performance of seven combination methods was compared on ACPs740 (as training datasets), and ACPs164 (as an independent validation dataset).

Table 1 shows the impact of different combinations of hyperparameters on the independent validation dataset ACPs164, and we first determine the parameter combination based on accuracy, and then select the final hyperparameters based on the values of Matthews correlation coefficient (MCC), Sensitivity (SE), and Specificity (SP). As shown in Table 1, the best metric values are highlighted in bold. It is observed that R generally performs optimally when set to 1.0, and the number of neurons in the hidden layer is typically chosen as 256 or 512. Based on comprehensive metrics such as accuracy (ACC) and MCC, we selected the hyperparameter combination c3 that yielded the best overall performance, namely R = 1.0, C = 256, and D = 512.

The selection of a suitable pre-trained BERT model is crucial in the feature channel of chemical Bert. We compared two different BERT models, namely chemBERTa [59] based on the robustly optimized BERT pre-training approach (RoBerta) [60] and BERT-base [53,54], for feature extraction of our SMILES-formatted data. chemBERTa was pre-trained on a large amount of SMILES-formatted data, while BERT-base was pre-trained on human natural language text. For chemBERTa, we also compared two different tokenization methods, namely SMILES-tokenizer (ST) and BPE’s tokenizer (BT) [61]. Table 2 shows the results of our model validation on independent validation dataset ACPs164. The experimental results show that these BERT models exhibit trivial performance differences, but the chemBERTa model based on the SMILES tokenizer achieves better results.

### 2.3. Comparison of Fused Features

**Figure 3:**
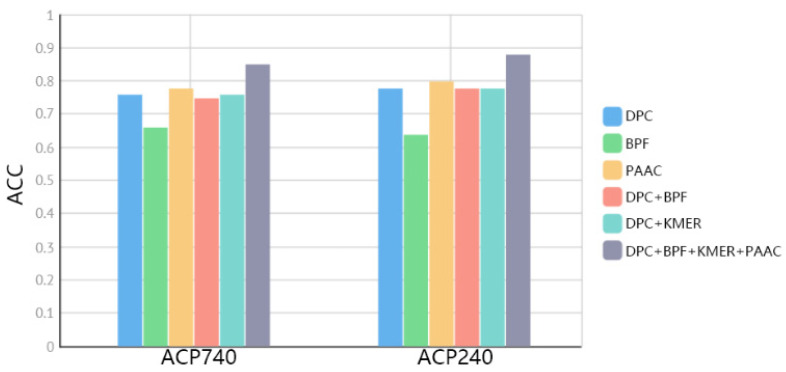
Comparison of fused features on ACP740 and ACP240 datasets.

The third channel of the ACP-BC model consists of four manually selected features. These four features include composition information [34,38,43,47,49], local information [41,43,47], long-range relational information [23], and locally calculated information based on protein physicochemical properties [35,45]. To investigate the impact of these features, we conducted a series of ablation experiments on the ACP740 and ACP240 datasets to evaluate the integration effect. Firstly, we conducted individual experiments for each feature representation method, and the results are shown in Figure 3. PAAC outperformed other features, but as the other features are extracted from different perspectives, their quality cannot be simply evaluated. Therefore, we designed experiments combining BERT + LSTM with other features. Specifically, we designed the following experiments: BERT + LSTM + DPC, BERT + LSTM + BPF,BERT + LSTM + DPC + BPF, BERT + LSTM + BPF + DPC + KMER, and BERT + LSTM + DPC + BPF + KMER + PAAC. From Figure 3, it can be observed that these features represent different levels of information, and thus achieve better results when fused together.

### 2.4. Comparison of Existing Methods

To demonstrate the effectiveness of our designed model, we compared ACP-BC with other state-of-the-art methods. We evaluated the performance of ACP-DA [47], ACP-DL [43], GRCI-Net [50], DeepACPpred [51], ACP-MHCNN [40], ACP-check [49], and other methods using the same ACP740 and ACP240 datasets for a fair comparison. Compared to ACP-DA, our model achieved better results with the combined use of two feature enhancement methods. In terms of feature engineering, the features we selected were more representative and capable of extracting amino acid sequence features from multiple perspectives. Additionally, compared to other studies, we proposed the novel application of SMILES sequences and the BERT model for ACPs recognition. Our experimental results showed promising outcomes. As shown in Table 3, our proposed method outperformed ACP-DA, ACP-DL, DeepACPpred, and other models in terms of performance on the ACP740 and ACP240 datasets. We achieved an accuracy of 0.87 and 0.90, respectively, surpassing other models. In terms of the MCC metric, our model surpassed most methods on the ACP740 dataset, while obtaining the best results on the ACP240 dataset. These initial results provide preliminary evidence of the effectiveness of our data augmentation methods. Specifically, on the ACP240 dataset, our method demonstrated improvements of 1.3% on ACC, 0.01 on MCC, and 0.01 on AUC compared to ACP-Check. This indicates that our model performs better on small datasets. For the ACP740 dataset, the performance of ACP-BC (SE, 0.87. SP, 0.88) is better than the ACP-DA, ACP-DL, GRCI-NET, and DeepACPpred, except ACP-MHCNN and ACP-CHECK. For the ACP240 dataset, the performance of ACP-BC (SE, 0.90. SP, 0.89) is better than the ACP-DA, ACP-DL, GRCI-NET, and DeepACPpred, except ACP-CHECK and ACP-DA. Overall, our designed model successfully extracted features at different levels and exhibited stronger compatibility with small datasets. Thus, our model possesses great potential for the prediction of ACPs and non-ACPs.

**Figure 4:**
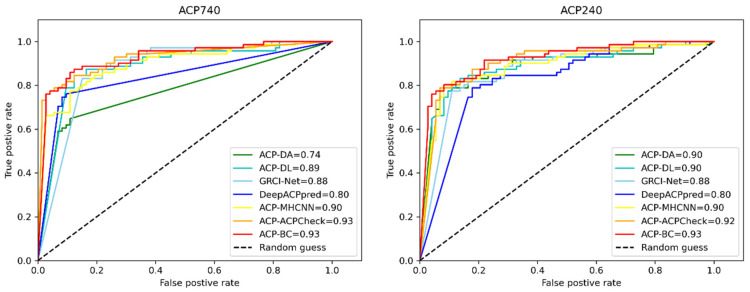
Comparison of ROC curves for ACPs prediction using different methods on ACP740 and ACP240 datasets.

In addition, to provide more insight into our achieved results, we also plotted receiver operating characteristic (ROC) curves to further demonstrate the performance of ACP-BC, as shown in Figure 4. The horizontal axis of the ROC curve is False Positive Rate, and the vertical axis is True Positive Rate. We constantly achieved high Area Under the Curve (AUC) values on ACP740 and ACP240 datasets, and they were both 0.93. The ROC of AUC is steeper than other compared models, encompassing other comparison methods such as ACP-DA, ACP-DL, GRCI-Net, etc., indicating that our model ACP-BC is superior to other models and has better stability. Although the AUC of ACP-Check on the ACP740 dataset is 0.93, which is the same as our ACP-BC, on the ACP240 dataset, the AUC of ACP-BC is higher than ACP-Check by 1%. This further indicates that our model performs better on small datasets after data augmentation. The improvement of AUC metrics on two datasets demonstrates the generality and effectiveness of our proposed method. Figure 4 shows that the ACP-BC outperformed the existing predictors on ACP740 and ACP240 datasets.

### 2.5. Independent Validation

To further validate the performance of the ACP-BC model, we conducted independent validations on the ACPred-Fuse dataset [62] and ACP20, respectively.

#### 2.5.1. Independently Validating on the ACPred-Fuse Dataset

**Figure 5:**
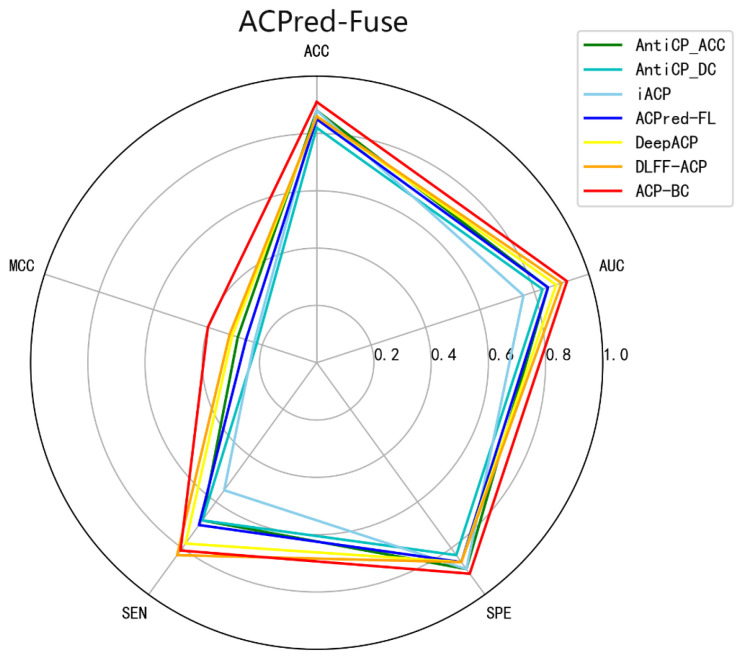
Comparison of different methods on ACPred-Fuse datasets.

To further validate the generality and feature mining capabilities of our proposed approach, based on feature fusion and ChemBERT, we applied this method to the ACPred-Fuse dataset. This dataset contains a substantial number of non-ACP compounds without anticancer activity, simulating real-world positive-to-negative sample ratios. Additionally, we compared our ACP-BC model with three machine learning models AntiCP [22], iACP [30], ACPred-FL [38] and two deep learning models (DLFF-ACP [44], DeepACP [52]. Detailed data is presented in Table 4, revealing that the ACP-BC model’s performance stands out with an ACC of 0.91, MCC of 0.40, specificity of 0.91, and an AUC of 0.92. In comparison to other methods, ACP-BC demonstrated improvements in ACC, MCC, specificity, and AUC ranging from 3% to 9%, 8% to 18%, 2% to 8%, and 2% to 16%, respectively. Its comprehensive performance clearly surpasses that of the other five models, further affirming the potential of our method in mining deep features from ACP sequences for accurate ACP identification. Figure 5 provides a more intuitive comparison.

#### 2.5.2. Independently Validating on the ACP20 Dataset

To further validate the effectiveness and robustness of the ACP-BC model, we conducted independent validation using the ACP20 dataset, which consists of 10 ACPs and 10 non-ACPs samples. Our model was not trained on this dataset, allowing us to better evaluate the model’s robustness. Through experiments, we found that when applying the model trained on the ACP740 dataset to the ACP20 dataset, all ACPs and non-ACPs samples were accurately identified. The independent validation results are shown in Table 5. The “Score” column represents the scores output by the model for the given protein sequences. Most ACPs samples obtained high scores, greater than 0.7. If the predicted score is greater than 0.5, the corresponding peptide is considered as ACPs, and if not, it is considered as non-ACPs. The independent validation results demonstrate that ACP-BC exhibits strong robustness and generalization, making it applicable for the identification of unknown protein sequences.

## 3. Discussion

Cancer is a prevalent and deadly disease, and its treatment has always been a long-standing challenge. Anticancer peptides have demonstrated potent anticancer activity, and distinguishing between anticancer and non-anticancer peptides is a crucial step in anticancer peptide research. In this study, we propose a novel anticancer peptide identification model called ACP-BC, which integrates multiple features including sequence information and chemical information. Extensive experiments have shown that our method achieves high accuracy and robustness, making it suitable for anticancer peptide identification. In the following sections, we analyze the reasons behind the improved performance of our model.

Firstly, we employ an enhanced data augmentation method to preprocess the dataset, randomly replacing, shuffling, reversing, or subsampling each amino acid residue in each sequence with a probability of *p*. Experimental results demonstrate that using *p* = 0.01 to augment the entire ACPs dataset effectively enhances the model’s performance and generalization by doubling the amount of data.

For feature extraction from protein sequence information, we utilize BI-LSTM, BERT, and manually selected features as three channels to effectively capture different hierarchical features of amino acid sequences. In the first channel, the entire protein sequence is encoded through an embedding layer, which is trained together with the entire model. The resulting embedded representation of the original sequence is then input into a three-layer bidirectional LSTM, and the output of the Bi-LSTM serves as the information for the first channel. Experimental findings suggest that setting the embedding layer and LSTM’s hidden neuron counts to 256 and 512, respectively, yields optimal results. In the second channel, we introduce the structural information of amino acids in an innovative way. We employ a BERT model pre-trained on SMILES sequences to extract deep abstract features. Initially, the original sequence is converted into a molecular structure representation, and then SMILES, a structured symbolic language, is utilized to simplify chemical molecular formulas. Subsequently, the obtained SMILES-formatted data is input into the BERT model for fine-tuning, resulting in chemical molecular formula features related to protein sequences. In the process of selecting detailed parameters for the BERT model, a series of experiments are conducted, ultimately choosing ChemBERTa with a SMILES tokenizer as the feature extractor. In the third channel, we optimize the feature engineering methods used in other studies. We combine BPF, DPC, and PAAC features as manually selected features, which extract positional information, compositional information, and local information of the protein sequences, respectively. Through a series of ablation experiments, we demonstrate the effectiveness of these manually selected features in capturing diverse aspects of the sequences. The three channels of feature information complement each other, and their fusion enables better extraction of various hidden layers of information in protein sequences. To better fine-tune the BERT model, we employ multiple fully connected layers to integrate, abstract, and predict the extracted features. In the entire model, we assign a smaller learning rate to the BERT model than other layers by an order of magnitude, which is more suitable for fine-tuning training. In evaluating the effectiveness of our approach, we train and test our model on ACP740 and ACP240 datasets, and validate it on an independent ACP20 dataset [19,45,62]. Experimental results demonstrate that our designed model achieves excellent performance, surpassing other models in multiple metrics such as ACC and MCC. It also performs exceptionally well on the independent dataset. In summary, our model exhibits great effectiveness, robustness, and generalization ability, and it can be readily applied to the identification of ACPs.

## 4. Materials and Methods

### 4.1. Dataset

An excellent dataset is crucial for establishing a reliable ACPs prediction model. In recent years, several excellent datasets have been established [19,30,38,43,45,62]. In this study, we utilized two benchmark datasets with large samples, ACP740 [30,36,43] and ACP240 [43], along with three independent validation datasets, ACP164 [38], ACPred-Fuse [62] and ACP20 [19,45,62]. The similarities between the four datasets are as follows: the ACPs verified in the experiment are used as positive samples, CD-HIT [63] is used to remove the peptide sequences with a similarity of more than 90%, and the antimicrobial peptides (AMPs) that do not have anticancer function are used as negative samples. These datasets are non-redundant and do not overlap, ensuring a solid foundation for our research. These datasets can be publicly accessed through the https://github.com/haichengyi/ACP-DL, http://server.malab.cn/ACPred-FL/, http://server.malab.cn/ACPred-Fuse and https://github.com/abcair/ACPNet, (accessed on 7 March 2023, China) Dataset Repository.

#### 4.1.1. Dataset ACP740

The ACP740 dataset was constructed by Yi et al. [43] from Chinese Academy of Sciences, Beijing, China; Chinese Academy of Sciences Xinjiang Technical Institute of Physics and Chemistry, Urumqi, Xinjiang, China. It consists of 364 positive samples (peptides with anticancer activity). Initially, 138 positive samples were collected from the study by Chen et al. [30], and 250 positive samples were collected from the study by Wei et al. The non-ACPs dataset, which contains 376 negative samples (peptides without anticancer activity), originally included 206 samples from Chen et al.’s study [30] and 170 samples from Wei et al.’s study [38]. To avoid dataset bias, we used the commonly used tool CD-HIT to remove 12 positive samples and 12 negative samples with a similarity exceeding 90%. As a result, we obtained the ACP740 dataset, which includes a total of 740 samples.

#### 4.1.2. Dataset ACP240

ACP240 is another dataset constructed by Yi et al. [43] from Chinese Academy of Sciences, Beijing, China; Chinese Academy of Sciences Xinjiang Technical Institute of Physics and Chemistry, Urumqi, Xinjiang, China. It consists of 129 positive samples (peptides with anticancer activity) and 111 negative samples (peptides without anticancer activity but with antimicrobial activity). To remove positive and negative samples with a similarity exceeding 90%, we also used the widely used CD-HIT. As a result, we obtained the ACP240 dataset, which includes a total of 240 samples.

#### 4.1.3. Independent Validation Dataset ACP164

To verify the generalization ability of the ACP-BC model, we selected the ACP164 [38] dataset as the independent validation set. The ACP164 dataset was constructed by Wei et al. from School of Computer Science and Technology, Tianjin University, Tianjin, China; Biomedicine Discovery Institute and Department of Biochemistry and Molecular Biology, Monash Centre for Data Science, Faculty of Information Technology, Monash University, Clayton, Australia; School of Computer Software, Tianjin University, Tianjin, China; State Key Laboratory of Medicinal Chemical Biology, Nankai University, Tianjin, China. It includes 82 experimental validated ACPs from the positive dataset, as well as the same number of non-ACPs from the negative dataset. All peptides did not appear in the training dataset, ensuring a fair evaluation of model performance.

#### 4.1.4. Independent Validation Dataset ACPred-Fuse

To further validate the generality and feature mining capability of our proposed method, which is based on feature fusion and ChemBERT, we selected the ACPred-Fuse dataset for training and testing. The ACPred-Fuse dataset was collected by Rao et al. [62] from School of Mechanical Electronic & Information Engineering, China University of Mining & Technology, Beijing, China; School of Software, College of Intelligence and Computing, Tianjin University, Tianjin, China; School of Software, Shandong University, Jinan, China. The ACPred-Fuse dataset with positive samples from Chen et al. [30], Tyagi et al. [9], and the ACP database CancerPPD [64], while negative samples were from Swiss Prot [65]. To avoid the impact of data bias, we used the CD-HIT tool to exclude peptide sequences with positive and negative similarities greater than 0.8. The final dataset consisted of 3210 samples, including 332 positive samples and 2878 negative samples. This dataset contains a large number of negative samples, simulating a real situation where there are far more negative samples than positive samples. By introducing a large number of negative samples, we have verified that our method can mine the deep abstract features of ACP sequences.

#### 4.1.5. Independent Validation DatasetACP20

ACP20 is a dataset constructed by Sun et al. [45] from Key Laboratory of Symbol Computation and Knowledge Engineering of Ministry of Education, College of Computer Science and Technology, Jilin University, Changchun, China; School of Computer Science and Artificial Intelligence Aliyun School of Big Data School of Software, Changzhou University, Changzhou, China; College of Software, Jilin University, Changchun, China. It consists of 10 positive samples (active anticancer peptides) labeled as ACPs and 10 negative samples (non-active peptides) labeled as non-ACPs.

To better train the model, we divided the dataset into training and testing sets. ACP740 and ACPC240 datasets were randomly shuffled and split, with 80% used for training and 20% for testing. ACP164, ACPred-Fuse and ACP20 were used as an independent validation set to further verify the model’s generalization ability. Notably, all existing methods will be evaluated on the test set for fair evaluation.

### 4.2. Data Augmentation

In gene sequences, mutations and alternative splicing are common phenomena. To improve model performance and robustness, data augmentation methods such as random replacement and random insertion are frequently employed in sequence analysis. In this study, we utilized a combination of augmentation techniques, referred to as Combining Augmentations [66].

#### 4.2.1. Replacement

Randomly replace amino acid residues in the sequence. For a sequence S with a length of n amino acids, we replace amino acids in the sequence with a probability *p*, substituting them with the closest amino acid. Here, p is a hyperparameter designed for this purpose. The closest amino acids are determined based on the structural and physicochemical properties, and the pairing scheme used includes ((A,V), (S,T), (F,Y), (K,R), (C,M), (D,E), (N,Q), (V,I)).

#### 4.2.2. Local Random Shuffling

Randomly select a subsequence of length t from the sequence and insert it into other positions. Although this disrupts the sequence structure to a great extent, it allows the model to focus more on permutation-invariant features.

#### 4.2.3. Sequence Reversion

Reverse the amino acid sequence. For a sequence S [s_1_, s_2_, …, s_n−1_, s_n_] with a length of n amino acids, we reverse it to obtain S’ [s_n_, s_n−1_, …, s_2_, s_1_]. Protein sequences have a directional orientation, ranging from the N-terminus to the C-terminus. Reversing the protein sequence changes the overall structure and function of the protein. However, the reversed sequence may encourage the model to effectively utilize short-range features, focus more on local information, and be more suitable for LSTM models.

#### 4.2.4. Combining Augmentations

Two strategies are employed: single augmentation and combined augmentation. Single augmentation involves selecting one augmentation method, while combined augmentation combines two augmentation methods.

### 4.3. Encoding and Embedding Representations of Amino Acid Sequences


(1) $$P=p_{1}p_{2}p_{3}\ldots p_{n}$$

In the equation, P represents a peptide sequence [42], where $p_{1}$ denotes the first amino acid of the peptide, $p_{2}$ denotes the second amino acid, $p_{3}$ denotes the third amino acid, and $p_{n}$ represents the *nth* amino acid in the peptide sequence. Each amino acid corresponds to a letter in the standard amino acid alphabet. The standard amino acid alphabet consists of 20 letters: A, C, D, E, F, G, H, I, K, L, M, N, P, Q, R, S, T, V, W, and Y. To encode peptide sequences into numerical vectors, four different types of features are chosen: BPF, DPC, PAAC, and the k-mer sparse matrix feature. Each feature is described as follows.

#### 4.3.1. BPF

Binary profiles features [22] can differentiate peptides that are chemically similar but functionally distinct. However, due to the varying lengths of peptides, it is challenging to establish a fixed-length pattern. To address this issue and generate fixed-length patterns, we employ a binary one-hot vector encoding method [38]. Since there are 20 amino acids in the amino acid alphabet, each amino acid is represented by a binary one-hot vector of length 20 to ensure consistent feature length. For example, the first letter “A” in the amino acid alphabet is represented by the numerical vector $f\left(A\right)=[1,0,0,\ldots ,0]$. The second letter “C” representing the amino acid is represented by the numerical vector $f\left(C\right)=[0,1,0,\ldots ,0]$, and so on. Finally, the last letter “Y” representing the amino acid is represented by the numerical vector $f\left(Y\right)=[0,0,0,\ldots ,1]$. Therefore, the expression for Binary Profile Feature (BPF) is given as:

(2) $$F\left(\mathrm{BPF}[K]\right)=[f(p1),\;f(p2),\;f(p3),\ldots ,\;f(\mathrm{pK})]$$

where K represents the length of peptides similar to the N-terminal amino acid. Experimental results indicate that for optimal performance of ACP-DL and ACP-DA, selecting the first 7 letters to represent BPF (k = 7) yields the best results.

#### 4.3.2. DPC

Dipeptide Composition (DPC) [21] represents the percentage composition of the 400 possible dipeptides formed by the 20 amino acids. Unlike Amino Acid Composition (AAC), DPC considers the combinations of adjacent amino acids, thereby capturing additional information about the local arrangement of peptides/proteins. In biology, statistical analysis of dipeptide frequencies is also a common classification method. The expression for DPC is as follows:

(3) $$f\left(a,b\right)=\frac{\mathrm{Nab}}{(N\;-\;1)},\;a,\;b\in \{A,\;C,\;D,\ldots Y\}$$

where Nab represents the number of dipeptides composed of amino acids of types “a” and “b”, while N represents the length of the peptide sequence.

#### 4.3.3. PAAC

Pseudo Amino Acid Composition (PAAC) [28] expands the concept of amino acid composition by combining sequence order information. The feature extraction method of PAAC generates a feature vector of 20 + λ dimensions, where the first 20 dimensions represent amino acid composition features, and the additional λ dimensions capture the sequence order information. Here, λ is a hyperparameter that can be adjusted based on the input information’s dimensionality. The sequence order effect can be represented by a set of correlation factors, which θ are defined as follows:

(4) $$\left\{\begin{array}{l} \begin{array}{l} \theta_{1}=\frac{1}{L\;-\;1}\sum_{i=1}^{L\;-\;1}\;\Theta \left(p_{i},\;p_{i+1}\right)\\ \theta_{2}=\frac{1}{L\;-\;2}\sum_{i=1}^{L\;-\;2}\;\Theta \left(p_{i},\;p_{i+2}\right)\\ \theta_{3}=\frac{1}{L\;-\;3}\sum_{i=1}^{L\;-\;3}\;\Theta \left(p_{i},{\;p}_{i+3}\right),\left(\lambda <L\right)\\ \;\;\;\;\;\;\;\ldots \end{array}\\ \theta_{3}=\frac{1}{L\;-\;3}\sum_{i=1}^{L\;-\;3}\;\Theta \left(p_{i},\;p_{i+3}\right) \end{array}\right.$$

In the above equation, $\theta_{1}$ represents the first-level correlation factor between two adjacent amino acids, $\theta_{2}$ represents the second-level correlation factor between two amino acids separated by one position, and so on. The calculation of the correlation function in the formula is as follows:

(5) $$\Theta \left(p_{i},p_{j}\right)=\frac{1}{3}\left\{{\left[H_{1}\left(p_{j}\right)-H_{1}\left(p_{i}\right)\right]}^{2}+{\left[H_{2}\left(p_{j}\right)\;-\;H_{2}\left(p_{i}\right)\right]}^{2}\right\}$$

In the above equation, $H_{1}\left(p_{i}\right)$ and $H_{2}\left(p_{i}\right)$ represent the hydrophobicity value and hydrophilicity value, respectively. In practical applications, additional physicochemical properties can also be used for calculation. The sequence order effect of a protein can be reflected to some extent by a set of sequence correlation factors $\theta_{1},\theta_{2}$, $\theta_{3}$… Therefore, the amino acid composition can be expanded to 20 + λ dimensions.

(6) $$X=\left[x_{1},\;x_{1},\cdots ,{\;x}_{20+1},\;\cdots ,{\;x}_{20+\lambda }\right]$$

Each Xu represents:

(7) $$x_{u=}\left\{\begin{array}{l} \frac{f_{u}}{\sum_{i=1}^{20}\;f_{i}+w\sum_{j=1}^{\lambda }\;\theta_{j}}\;\;\left(1\;\leq \;u\;\leq \;20\right)\\ \frac{w\theta_{u\;-\;20}}{\sum_{i=1}^{20}\;f_{i}+w\sum_{j=1}^{\lambda }\;\theta_{j}}\;\;\left(20+1\;\leq \;u\;\leq \;20+\lambda \right) \end{array}\right.$$

In the above equation, $f_{i}$ represents the normalized occurrence frequency of the 20 amino acids in the X amino acid sequence, and w is the weight factor for the sequence order effect, with a default value of 0.05. $\theta_{j}$ is the j-tier sequence correlation factor for the amino acid sequence X. The vector of u from 1 to 20 dimensions is mainly determined by the composition characteristics of amino acids, while the vector of u from 20 + 1 to 20 + λ dimensions is mainly determined by the values of related factors. The resulting vector of 20 + λ dimensions is referred to as the Pseudo-Amino Acid Composition.

#### 4.3.4. K-mer Sparse Matrix

The k-mer sparse matrix was proposed by You et al. in 2016 [42]. K-mer refers to scanning a sequence from left to right and extracting K consecutive elements using a sliding window. In the amino acid sequence, we initially divide the 20 amino acids into 7 groups based on their dipole moments and side chain volumes: (A, G, V), (I, L, F, P), (Y, M, T, S), (H, N, Q, W), (R, K), (D, E), and (C). Therefore, the amino acid sequence is transformed into a sequence composed of 7 letters representing the groups, such as a, b, c, and so on. By considering not only the properties of a single amino acid and its preceding K−1 amino acids, but also treating any K consecutive amino acids as a unit, the sequence is scanned from left to right, and K-mer words are extracted to represent the characteristics of the amino acid sequence. If the length of the peptide chain is L, there could be $7^{k}$ different K-mers, and a total of L−K+1 scanning steps are performed, resulting in a generated two-dimensional matrix. There are a total of L−K+1 different $7^{k}$ K-mers, resulting in a generated two-dimensional matrix through L−K+1 scanning steps. The values in the sparse matrix can be summarized as follows:

(8) $$M=\left(a_{ij}\right)7^{k}\times \left(L-K+1\right)$$

(9) $$a_{\mathrm{ij}}=\left\{\begin{array}{c} 1,\\ 0, \end{array}\begin{array}{c} \mathrm{if}\;Q_{j}Q_{j+1}{\ldots Q}_{j+k-1}=\mathrm{Kmer}(i)\\ \mathrm{else} \end{array}\right.$$

where kmer(i) represents the i-th k-mer. Each column of matrix M is a unit vector where only one element is 1, and the remaining elements are 0.

### 4.4. Bi-LSTM

The bidirectional long short-term memory network (Bi-LSTM) is a commonly used type of recurrent neural network (RNN) [51] that has been widely applied in natural language processing (NLP), speech recognition, and machine translation. Bi-LSTM consists of two LSTM networks, one processing the input sequence from left to right and the other processing it from right to left. The outputs of the two networks are concatenated to form the final output. Since the residues in a peptide sequence are influenced by both preceding and succeeding residues, similar to how the order of words in a sentence affects its meaning, we choose Bi-LSTM to capture the bidirectional information in the sequence.

### 4.5. Chemical BERT

To better distinguish between ACPs and non-ACPs, we introduced the BERT pre-training model [54] to explore deeper-level features. BERT is a transformer-based contextual language representation model that has demonstrated outstanding performance in numerous natural language processing (NLP) tasks. It employs a pre-training and fine-tuning structure, starting with extensive unsupervised training on unlabeled data for general understanding and then fine-tuning for specific tasks. Using BERT for pre-training along with task-specific fine-tuning has shown better results compared to previous methods in many NLP tasks. Over the past two years, BERT has become a widely used approach for learning text feature representations through self-supervision. Many researchers have utilized BERT to represent biological or biomedical information, such as promoter recognition prediction [67], among other aspects.

Unidirectionality is a significant issue in sequence processing models as it limits the flexibility of the entire model structure during training. Due to the limited memory capacity of Bi-LSTM, it tends to focus more on neighboring information. In contrast, BERT effectively utilizes both forward and backward information through contextual pre-training, enabling better capturing of the global relationships between amino acids. Therefore, we employ BERT to complement the deep abstract features required for predicting amino acid sequences.

**Figure 6:**
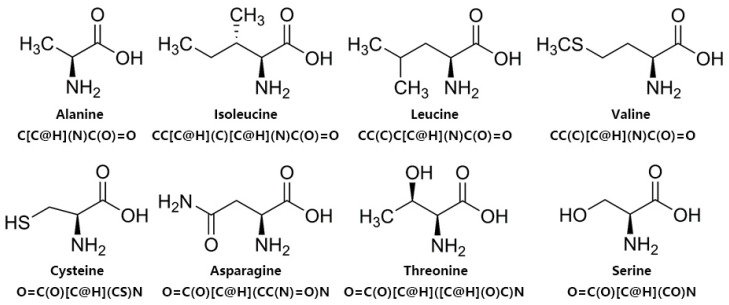
Molecular structures of amino acids and their corresponding SMILES sequence representations.

To better represent the structural information of amino acid sequences, we utilize the SMILES sequences in practical applications. Firstly, we convert the amino acid sequences back to molecular structures and then represent these structures using a specific markup language specification. An example of a simple structure is dinitrogen, with the representation N ≡ N (N#N). In Figure 6, we illustrate the molecular structures of some amino acids and their corresponding SMILES representations. For instance, the SMILES sequence for Alanine is C[C@H](N)C(O)=O, and for Isoleucine, it is CC[C@H](C)[C@H](N)C(O)=O. By utilizing SMILES sequences to represent amino acid sequences, we can better capture the structural information of the entire peptide chain. Combining the representation of SMILES sequences with the powerful capability of BERT in extracting deep abstract information, we employ ChemBERT [59]. It is a BERT model trained on a large number of molecular SMILE sequences, which is integrated into our model for fine-tuning to better extract abstract deep features from ACP sequences.

### 4.6. Performance Evaluation

The ultimate goal of the ACP-BC model is to effectively identify ACPs and non-ACPs, which is a binary classification problem. We selected six commonly used evaluation metrics for binary classification, including Accuracy (ACC), Sensitivity (SE), Specificity (SP), Matthews correlation coefficient (MCC), and area under ROC curve (AUC) [40]. The formulas for these metrics are as follows:

(10) $$\mathrm{Accuracy}\left(\mathrm{ACC}\right)=\frac{\mathrm{TP}+\mathrm{TN}}{\mathrm{TP}+\mathrm{TN}+\mathrm{FP}+\mathrm{FN}}$$

(11) $$\mathrm{Sensitivity}\left(\mathrm{SE}\right)=\frac{\mathrm{TP}}{\mathrm{TP}+\mathrm{FN}}$$

(12) $$\mathrm{Specificity}\left(\mathrm{SP}\right)=\frac{\mathrm{TN}}{\mathrm{TN}+\mathrm{FP}}$$

(13) $$\mathrm{Matthews}\;\mathrm{correlation}\;\mathrm{coefficient}\;(\mathrm{MCC})=\frac{\mathrm{TP}\;\times \;\mathrm{TN}\;-\;\mathrm{FP}\times \mathrm{FN}}{\sqrt{(\mathrm{TP}+\mathrm{FN})\;\times \;(\mathrm{TP}+\mathrm{FP})\times (\mathrm{TN}+\mathrm{FP})\;\times \;(\mathrm{TN}+\mathrm{FN})}}$$

where TP represents true positive, indicating the correct prediction of ACPs as ACPs in the peptide sequence, TN represents true negative, indicating the correct prediction of non-ACPs as non-ACPs in the peptide sequence, FP represents false positive, indicating the incorrect prediction of ACPs as non-ACPs in the peptide sequence, and FN represents false negative, indicating the incorrect prediction of non-ACPs as ACPs in the peptide sequence. The precision (P) is calculated as TP/(TP + FP), and the recall (R) is calculated as TP/(TP + FN).

## 5. Conclusions

In this study, we proposed a model aimed at accurately identifying anticancer peptides by Bi-LSTM and chemical information, called ACP-BC, which is a two-class classification problem. To find useful features, we compare the performance of an autoencoder feature, a newly proposed chemical molecular feature, and four commonly used feature combinations on two benchmark datasets. The experimental results demonstrate that the combination of these six features plays a positive role in ACPs and non-ACPs identification. Finally, we employ a fully connected network to handle the feature combinations for ACPs recognition.

Comparing with six existing state-of-the-art methods, ACP-BC shows improvements in various performance metrics, including ACC, MCC, SE, SP, and AUC. ACP-BC also exhibits improved performance metrics on the ACP740 dataset. When tested on an independent dataset ACP20, ACP-BC accurately predicts all 10 ACPs samples. Through a series of experiments, we demonstrate the effective and accurate identification of ACPs and non-ACPs by ACP-BC. However, our proposed method still has limitations and we are still unable to accurately identify certain anticancer peptides. There are many reasons for this phenomenon. On the one hand, this may be a problem with the dataset. Some samples of anticancer peptides are relatively unique and have significant distribution differences compared to other samples, making it difficult for the model to identify them based on experience. On the other hand, it may be that our model itself needs improvement and fails to accurately extract more effective and recognizable features, resulting in the model being unable to accurately recognize anticancer peptides. In following research work, more datasets can be introduced and combined with new machine learning methods, such as contrastive learning, to reduce the impact of non real negative samples on model performance. At the same time, we try to gain a deeper understanding of anticancer peptides and use methods that are more suitable for extracting anticancer peptides features, in order to identify anticancer peptides more accurately. In future work, we plan to incorporate more complex and effective features and deploy them on a network to develop an intelligent system for accurate ACPs identification.

## Figures and Tables

**Table 1:** Selection of hyperparameters on independent validation ACP164 datasets.

Combination	R	C	D	ACC	MCC	SE	SP
c1	1.0	128	256	0.72	0.44	0.75	0.70
c2	1.0	256	256	0.80	0.62	**0.88**	0.73
c3	1.0	256	512	**0.81**	**0.62**	0.84	0.78
c4	1.0	512	512	0.80	0.59	0.79	**0.80**
c5	2.0	128	256	0.76	0.52	0.77	0.79
c6	2.0	256	512	0.80	0.60	0.83	0.76
c7	2.0	512	512	0.75	0.50	0.76	0.74

Note: The maximum value is marked in bold. ACC: accuracy. MCC: Matthews correlation coefficients. SE: sensitivity. SP: specificity.

**Table 2:** Performance comparison of BERT on ACP740 and ACP240 datasets.

Dataset	BERT	ACC	MCC	SE	SP
ACP740	ChemBerta + ST	**0.87**	**0.75**	0.87	0.88
	ChemBerta + BT	0.85	0.69	0.85	0.85
	BERT-base	0.86	0.70	0.85	0.86
ACP240	ChemBerta + ST	**0.90**	**0.90**	0.90	0.89
	ChemBerta + BT	0.86	0.72	0.87	0.85
	BERT-base	0.89	0.76	0.88	0.88

Note: The maximum value is marked in bold. ACC: accuracy. MCC: Matthews correlation coefficients. SE: sensitivity. SP: specificity.

**Table 3:** Comparison of existing methods on ACP740 and ACP240 datasets.

Dataset	Methods	ACC	MCC	SE	SP	AUC
ACP740	ACP-DA	0.81	0.58	0.80	0.82	0.74
	ACP-DL	0.81	0.62	0.81	0.80	0.89
	GRCI-Net	0.82	0.65	0.84	0.82	0.88
	DeepACPpred	0.85	0.71	0.85	0.85	0.80
	ACP-MHCNN	0.86	0.72	**0.89**	0.83	0.90
	ACPCheck	0.87	0.75	0.86	0.88	0.93
	ACP-BC (ours)	**0.87**	**0.75**	0.87	**0.88**	**0.93**
ACP240	ACP-DA	0.89	0.78	0.88	0.89	0.90
	ACP-DL	0.84	0.68	0.88	0.78	0.90
	GRCI-Net	0.88	0.75	0.89	0.88	0.88
	DeepACPpred	0.86	0.72	0.88	0.84	0.80
	ACP-MHCNN	0.83	0.67	0.90	0.76	0.90
	ACPCheck	0.89	0.77	**0.91**	0.85	**0.92**
	ACP-BC (ours)	**0.90**	**0.78**	0.90	**0.89**	0.93

Note: The maximum value is marked in bold. ACC: accuracy. MCC: Matthews correlation coefficients. SE: sensitivity. SP: specificity. AUC: area under curve.

**Table 4:** Comparison of different methods on ACPred-Fuse dataset.

Dataset	Methods	ACC	MCC	SE	SP	AUC
	AntiCP_ACC	0.88	0.29	0.68	0.89	0.85
	AntiCP_DC	0.82	0.22	0.68	0.83	0.83
	iACP	0.88	0.23	0.55	0.89	0.76
ACPred-Fuse	ACPred-FL	0.85	0.26	0.70	0.86	0.85
	DeepACP	0.86	0.31	0.78	0.86	0.88
	DLFF-ACP	0.86	0.32	0.83	0.86	0.90
	ACP-BC (ours)	**0.91**	**0.40**	0.81	**0.91**	**0.92**

Note: The maximum value is marked in bold. ACC: accuracy. MCC: Matthews correlation coefficients. SE: sensitivity. SP: specificity. AUC: area under curve.

**Table 5:** The results on independent validation datasets.

Sequence	Score	Lable
KLWKKIEKLIKKLLTSIR	0.85	ACPs
YIWARAERVWLWWGKFLSL	0.88	ACPs
DLFKQLQRLFLGILYCLYKIW	0.82	ACPs
AIKKFGPLAKIVAKV	0.68	ACPs
RWNGRIIKGFYNLVKIWKDLKG	0.93	ACPs
KVWKIKKNIRRLLHGIKRGWKG	0.73	ACPs
GFWARIGKVFAAVKNL	0.78	ACPs
AFLYRLTRQIRPWWRWLYKW	0.78	ACPs
RIWGKHSRYIKIVKRLIQ	0.92	ACPs
QIWHKIRKLWQIIKDGF	0.67	ACPs
CGESCVWIPCVTSIFNCKCKENKVCYHDKIP	0.16	non-ACPs
SDEKASPDKHHRFSLSRYAKLANRLANPKLLETFLSKWIGDRGNRSV	0.18	non-ACPs
DVKGMKKAIKGILDCVIEKGYDKLAAKLKKVIQQLWE	0.10	non-ACPs
AGWGSIFKHIFKAGKFIHGAIQAHND	0.03	non-ACPs
ATCDLASGFGVGSSLCAAHCIARRYRGGYCNSKAVCVCRN	0.05	non-ACPs
GWKIGKKLEHHGQNIRDGLISAGPAVFAVGQAATIYAAAK	0.36	non-ACPs
FLGALIKGAIHGGRFIHGMIQNHH	0.25	non-ACPs
FLPAIAGILSQLF	0.40	non-ACPs
ALWMTLLKKVLKAAAKALNAVLVGANA	0.12	non-ACPs
EGGGPQWAVGHFM	0.29	non-ACPs

## Data Availability

Publicly available datasets were analyzed in this study. Codes and data are available here: https://github.com/shunmengfan/ACP-BC/tree/master (accessed on 1 August 2023).

## Abbreviations

ACPs	Anticancer peptides	
non-ACPs	Non-Anticancer peptides	
AAC	amino acid composition	
DPC	dipeptide composition	
BPF	binary profiles feature	
PAAC	pseudo-amino acid composition	
g-gap DPC	g-gap dipeptide composition	
RBF	radial basis function	
ATC	atomic composition	
RAAAC	reduced amino acid alphabet composition	
CTD	composition-transition-distribution	
QSO	quasi-sequence-order	
AAIF	amino acid index	
GAAC	grouped amino acid composition	
Am-PAAC	amphiphilic pseudo amino acid composition	
OPF	overlap property feature	
TOBF	twenty-one-bit feature	
AKDC	daptive skip dipeptide composition	
CNN	convolutional neural networks	
GCN	graph convolutional networks	
SVM	support vector machine	
RF	Random Forest	
PNN	probability neural network	
GRNN	generalized regression neural network
KNN	k-nearest neighbors
RNN	recurrent neural network
LSTM	long short-term memory
CKSAAGP	k-spaced amino acid group pairs
Bi-LSTM	bidirectional long short-term memory network
NLP	natural language processing
SMILES	Simplified Molecular Input Line Entry System
BERT	bidirectional encoder representation transformer
RoBerta	robustly optimized BERT pre-training approach
BPE	byte pair encoding
ChemBerta + ST	ChemBERTa with a SMILES tokenizer
ChemBerta + BT	ChemBERTa with a BPE’s tokenizer
TP	True Positive
FP	False Positive
TN	True Negative
FN	False Negative
ACC	accuracy
MCC	Matthews correlation
SE	sensitivity
SP	specificity
AUC	area under curve
ROC	receiver operating characteristic
